# Peripheral Inflammatory Markers in Autism Spectrum Disorder and Attention Deficit/Hyperactivity Disorder at Adolescent Age

**DOI:** 10.3390/ijms241411710

**Published:** 2023-07-20

**Authors:** Nikola Ferencova, Zuzana Visnovcova, Igor Ondrejka, Igor Hrtanek, Iveta Bujnakova, Veronika Kovacova, Andrea Macejova, Ingrid Tonhajzerova

**Affiliations:** 1 Biomedical Centre Martin, Jessenius Faculty of Medicine in Martin, Comenius University in Bratislava, 03601 Martin, Slovakia; nikola.ferencova@uniba.sk (N.F.); zuzana.visnovcova@uniba.sk (Z.V.); 2 Psychiatric Clinic, Jessenius Faculty of Medicine in Martin, Comenius University in Bratislava, University Hospital Martin, 03601 Martin, Slovakia; igor.ondrejka@uniba.sk (I.O.); igor.hrtanek@uniba.sk (I.H.); kovacova400@uniba.sk (V.K.); macejova5@uniba.sk (A.M.); 3 Society to Help People with Autism (SPOSA-Turiec), 03601 Martin, Slovakia; ibujnakova@gmail.com; 4 Department of Physiology, Jessenius Faculty of Medicine in Martin, Comenius University in Bratislava, 03601 Martin, Slovakia

**Keywords:** cytokines, autism spectrum disorder, attention deficit/hyperactivity disorder, adolescent age, receiver-operating characteristic curve analysis

## Abstract

Autism spectrum disorder (ASD) and attention deficit/hyperactivity disorder (ADHD) are associated with immune dysregulation. We aimed to estimate the pro- and anti-inflammatory activity/balance in ASD and ADHD patients at a little-studied adolescent age with respect to sex. We evaluated 20 ASD patients (5 girls, average age: 12.4 ± 1.9 y), 20 ADHD patients (5 girls, average age: 13.4 ± 1.8 y), and 20 age- and gender-matched controls (average age: 13.2 ± 1.9 y). The evaluated parameters included (1) white blood cells (WBCs), neutrophils, monocytes, lymphocytes, platelets, platelet distribution width (PDW), mean platelet volume, and derived ratios, as well as (2) cytokines—interferon-gamma, interleukin (IL)-1α, IL-1β, IL-2, IL-4, IL-6, IL-8, and IL-10, tumor necrosis factor-alpha (TNF-α), and derived profiles and ratios. ASD adolescents showed higher levels of WBC, monocytes, IL-1α, IL-1β, IL-2, IL-4, IL-6, IL-8, and IL-10, macrophages (M)1 profile, and anti-inflammatory profile than the controls, with ASD males showing higher monocytes, IL-6 and IL-10, anti-inflammatory profile, and a lower T-helper (Th)1/Th2+T-regulatory cell ratio than control males. The ADHD adolescents showed higher levels of PDW, IL-1β and IL-6, TNF-α, M1 profile, proinflammatory profile, and pro-/anti-inflammatory ratio than the controls, with ADHD females showing a higher TNF-α and pro-/anti-inflammatory ratio than the control females and ADHD males showing higher levels of IL-1β and IL-6, TNF-α, and M1 profile than the control males. Immune dysregulation appeared to be different for both neurodevelopmental disorders in adolescence.

## 1. Introduction

Autism spectrum disorder (ASD) and attention deficit/hyperactivity disorder (ADHD) represent frequently diagnosed neurodevelopmental disorders with onset typically occurring in childhood and persisting across the lifespan [1,2,3]. ASD is characterized by social communication deficits, the presence of repetitive and stereotyped behaviors, and restricted interests [4]. ADHD is characterized by inappropriate and persisting levels of inattentiveness, hyperactivity, and/or impulsivity [4]. It is well-accepted that core ASD and ADHD features, such as inattentiveness and social deficits, overlap, and that these overlapping patterns can be found in ASD- and ADHD-linked cognitive and behavioral traits [5,6,7,8]. Although both disorders frequently co-occur [9], their precise etiology and pathophysiology are still poorly understood. Among the mechanisms that may be involved in ASD and ADHD pathophysiologies, there has been increasing interest in the dysregulation of the immune system [10,11]. More specifically, the dysregulation of the peripheral and central immune systems, as well as the existence of the peripheral immune system and the central nervous system (CNS) cross-talk that may represent a crucial pathway in the development of a neuroinflammatory milieu contributing to ASD and ADHD pathophysiology [12,13].

The ongoing inflammatory process in the body can be simply revealed by frequently used complete blood count (CBC) tests. A CBC commonly includes red blood cells (RBCs), hemoglobin, hematocrit, RBC indices, such as RBC distribution width (RDW), white blood cells (WBCs) with differentials (i.e., neutrophils, lymphocytes, monocytes, eosinophils, and basophils), and platelets and platelet indices, including platelet distribution width (PDW) and mean platelet volume (MPV) [14,15]. Inflammation-related markers determined from a CBC include WBCs, neutrophils, monocytes, lymphocytes, MPV, PDW, and various ratios, including the neutrophil-to-lymphocyte ratio (NLR), monocyte-to-lymphocyte ratio (MLR), lymphocyte-to-monocyte ratio (LMR), platelet-to-lymphocyte ratio (PLR), platelet-to-monocyte ratio (PMR), MPV-to-lymphocyte ratio (MPVLR), and MPV-to-platelet ratio (MPVPR) [16,17,18,19,20,21,22,23,24,25]. Despite an increasing number of studies evaluating CBC indices in ASD and ADHD children [26,27,28,29,30,31,32,33], findings regarding ASD and ADHD at adolescent age are scarce. In this context, while Hesapcioglu et al. [34] revealed significantly higher levels of monocytes and RDW to be associated with a significantly lower LMR in ASD adolescents compared to controls, the most recent meta-analysis revealed significantly increased levels of WBCs, neutrophils, monocytes, and NLR in ASD patients (age range: 3-22 years) compared to controls [35]. With respect to ADHD, Akıncı and Uzun [36] revealed significantly higher levels of WBCs, neutrophils, NLR, PLR, MPV, and PDW in ADHD children and adolescents compared to controls. Other studies reported a significantly higher MPV in ADHD children and adolescents compared to controls [37,38]. Önder et al. [39] found a significantly increased NLR and PLR in ADHD adolescents compared to controls. On the other hand, ADHD youths showed no significant differences in CBC parameters (including WBCs, RDW, platelets, PDW, MPV, NLR, PLR, and MLR) compared to controls [40]. According to these inconsistent results, future studies in this critical age period are warranted.

**Figure 1:**
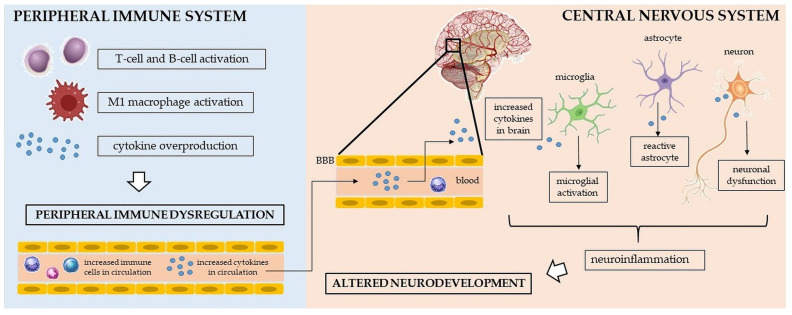
Several potential pathological mechanisms leading to dysregulation of the peripheral immune system and the central nervous system, resulting in altered neurodevelopment. BBB—blood–brain barrier.

Further, cytokines represent important cell-signaling molecules that regulate and influence immune responses at the periphery as well as in the CNS. More specifically, cytokines mediate the physiological signaling between immune and nonimmune cells and organize immune responses [41]. With respect to the CNS, cytokines are synthesized by neurons and glia, or they reach the CNS from the periphery through several pathways: 1. cytokines can pass via the blood–brain barrier (BBB)’s leaky regions; 2. cytokines can be actively transported via transport molecules, which are specific for individual cytokines; 3. cytokines can be transmitted via cranial nerves; and 4. cytokines can be released by transferred peripherally activated monocytes [42]. The presence of cytokines within the CNS is needed for appropriate autocrine and paracrine signaling and immune modulation [43]. However, the abnormal presence of cytokines in the CNS may contribute to neuroinflammation and consequent disturbances such as the disruption of neuronal plasticity or alterations in synaptic processes, resulting in altered brain development [44] (see Figure 1). Peripheral immune cells, including cytokines, can, thus, play a critical role in the neuroinflammation that seems to be involved in the etiopathogenesis of both neurodevelopmental disorders [44,45,46]; however, the precise mechanisms are still unknown.

With respect to ASD, the overproduction of proinflammatory cytokines peripherally as well as centrally has been repeatedly revealed in ASD children [47,48,49,50,51,52]. For example, significantly increased plasma levels of several cytokines, such as interleukin (IL)-1β, IL-6, IL-8, and IL-12p40, were found in ASD children compared to controls [47]. Another meta-analysis revealed significantly higher concentrations of IL-1β, IL-6, IL-8, interferon-gamma (IFN-γ), eotaxin, and monocyte chemoattractant protein-1 in ASD individuals compared to controls [53]. Increased levels of proinflammatory cytokines were also found within the CNS (specifically in brain tissues and cerebrospinal fluid) of ASD patients [54]. Persistent abnormal peripheral cytokine milieu in ASD patients may through the above-mentioned mechanisms contribute to the changes in cytokine levels within the CNS, thus, influencing ASD-related behavioral features [55,56]. With respect to ADHD, studies about inflammatory alterations have provided inconsistent findings. While most studies revealed higher levels of IL-6 in ADHD children [57,58,59,60], some other studies revealed no significant differences in the levels of IL-6 as well as IL-1β and tumor necrosis factor-alpha (TNF-α) between ADHD subjects and controls [61,62,63]. The recent meta-analysis reported significantly lower levels of TNF-α in ADHD children compared to controls with no between-group differences in levels of IL-1β and IL-6 [64]. On the other hand, the most recent meta-analysis including ADHD children, adolescents, and adults reported significantly higher levels of IL-6 and significantly lower levels of TNF-α in ADHD patients compared to controls [65].

On the other hand, the immune system also comprises regulatory elements that counter-regulate proinflammatory effects to maintain homeostasis, including anti-inflammatory cytokines, such as the IL-1 receptor antagonist (IL-1RA), IL-4, IL-10, and transforming growth factor-beta (TGF-β) [42,66,67]. However, studies regarding anti-inflammatory cytokines in ASD, as well as ADHD, reflect inconsistent findings. While one meta-analysis reported significantly decreased levels of TGF-β1 and borderline increased levels of IL-1RA [53], the more recent meta-analysis revealed decreased levels of IL-10 and IL-1RA, and slightly increased levels of IL-5 in ASD patients [68]. With respect to ADHD, the most studied IL-10 was shown to be increased, e.g., [57], or unchanged, e.g., [63], between ADHD and control subjects. No difference in IL-10 levels in ADHD patients compared to controls was confirmed by the recent meta-analysis [64]. According to the above-mentioned discrepancies in proinflammatory and anti-inflammatory cytokine activity, further research is needed to reveal the precise implication of individual cytokines in ASD and ADHD pathogeneses, especially during adolescence, being the critical age period of ongoing neuro- and immunodevelopment.

Further, both neurodevelopmental disorders exhibit a male bias in the prevalence rates, with a three times higher prevalence in males than in females for ASD [69] and two times higher prevalence in males than females for ADHD [70]. Similarly concerning the symptomatology, while ASD and ADHD males exhibit externalizing symptoms such as aggression [71,72,73], ASD and ADHD females exhibit more internalizing symptoms such as depression and anxiety [72,73]. However, the reasons underlying the ASD- and ADHD-linked sex biases remain unknown [74]. Theories of potential male vulnerability and/or female resilience/protection describe genetic, epigenetic, and environmental factors [74,75]. Immune activation in the CNS, including cytokine production and the activation of glial cells, may represent one of the possible mechanisms of the sex-dependent vulnerability to neurodevelopmental disorders [76]. With respect to ASD, a sex-specific inflammatory cytokine profile of the association between lower levels of IL-1β and IL-8 and higher symptom severity has been reported in ASD females, but not in ASD males [77]. Another study revealed distinct peripheral cytokine profiles in females (i.e., increased IL-1β, IL-7, and IL-12p40) and males (i.e., increased IL-1β, IL-3, IL-4, IL-5, IL-10, IL-12p40, IL-12p70, IL-18, and TNF-α) with Asperger syndrome [78]. Moreover, there is evidence for microglial dysregulation with a higher expression of ASD-elevated glial genes in the males’ than females´ cortexes [79], suggesting males´ higher susceptibility to neuroinflammatory-related pathways of ASD [74]. However, there is a lack of studies regarding microglial activity in ADHD. Concerning peripheral inflammatory markers in ADHD, Howard et al. revealed higher levels of WBCs, neutrophils, and NLR in ADHD females compared to ADHD males [36], and Chang et al. found a significant effect of sex on IL-1β, IL-17, and IFN-γ, but without a further analysis of sex differences [80]. Therefore, the need for the inclusion and analysis of both sexes in clinical research with a focus on the immune dysregulation of both neurodevelopmental disorders is highly warranted.

This study aimed to (1) evaluate peripheral proinflammatory and anti-inflammatory markers in ASD and ADHD with a focus on the rarely-studied adolescent age period with respect to sex, (2) assess cytokine ratios reflecting the balance of proinflammatory and anti-inflammatory activity in ASD and ADHD adolescents, an aspect which has not been studied yet, also with respect to sex, and (3) to determine potential biomarkers with predictive abilities for adolescent ASD and ADHD through a receiver-operating characteristic (ROC) curve analysis with an area under the curve (AUC) estimation. To the best of our knowledge, this is the first study comprehensively evaluating peripheral pro- and anti-inflammatory activity using multiple inflammatory markers as well as the proinflammatory/anti-inflammatory activity balance in both neurodevelopmental disorders (ASD and ADHD) with respect to sex at the adolescent age.

## 2. Results

### 2.1. Between-Group, between-Sex, and Mixed-Group × Sex Comparison of the Complete Blood Count Parameters and Selected Ratios

The effect of the group (ASD vs. ADHD vs. control group) was significant for the parameters WBCs, neutrophils, and PDW (F_[2]_ = 3.80, *p* = 0.028; F_[2]_ = 3.32, *p* = 0.043; F_[2]_ = 4.31, *p* = 0.018; respectively). There was no effect of the sex for all evaluated parameters. The effect of the group × sex interaction was significant for the parameters monocytes, the LMR, and MLR (F_[5]_ = 13.70, *p* = 0.018; F_[5]_ = 11.70, *p* = 0.039; F_[5]_ = 11.70, *p* = 0.039; respectively).

The post hoc analysis revealed that ASD adolescents were characterized by significantly higher levels of WBCs and monocytes compared to the control group (*p* = 0.042, *p* = 0.047, respectively). The post hoc analysis also revealed no significant differences between ASD females and control females. The post hoc analysis revealed that ASD males were characterized by significantly higher levels of monocytes compared to the control males (*p* = 0.033). The post hoc analysis also revealed that ADHD adolescents were characterized by a significantly higher PDW compared to the control group (*p* = 0.015). The post hoc analysis revealed no significant differences between either ADHD females and males or control females and males. No significant between-group difference was found in the remaining parameters. No significant between-sex difference within individual groups was found in all evaluated parameters. All results are summarized in Table 1 and Table 2.

### 2.2. Between-Group, between-Sex, and Mixed-Group × Sex Comparison of the Cytokines, Cytokine Profiles, and Cytokine Ratios

The effect of the group (ASD vs. ADHD vs. control group) was significant for the parameters IL-1α, IL-1β, IL-2, IL-4, IL-6, IL-8, IL-10, TNF-α, the M1 profile, proinflammatory profile, anti-inflammatory profile, Th1/Th2+Treg ratio, and proinflammatory/anti-inflammatory ratio (F_[2]_ = 12.25, *p* = 0.002; F_[2]_ = 17.14, *p* < 0.001; F_[2]_ = 17.18, *p* < 0.001; F_[2]_ = 8,50, *p* < 0.001; F_[2]_ = 29.42, *p* < 0.001; F_[2]_ = 14.19, *p* < 0.001; F_[2]_ = 15.39, *p* < 0.001; F_[2]_ = 16.10, *p* < 0.001; F_[2]_ = 28.14, *p* < 0.001; F_[2]_ = 6.31, *p* = 0.004; F_[2]_ = 16.58, *p* < 0.001; F_[2]_ = 7.75, *p* = 0.001; F_[2]_ = 7.76, *p* = 0.001; respectively). There was no effect of the sex for all evaluated parameters. The effect of the group × sex interaction was significant for the parameters IL-1α, IL-1β, IL-2, IL-6, IL-8, IL-10, the M1 profile, anti-inflammatory profile, Th1/Th2+Treg ratio, and proinflammatory/anti-inflammatory ratio (F_[5]_ = 17.27, *p* = 0.004; F_[5]_ = 18.08, *p* = 0.003; F_[5]_ = 21.91, *p* < 0.001; F_[5]_ = 31.49, *p* < 0.001; F_[5]_ = 16.72, *p* = 0.005; F_[5]_ = 16.85, *p* = 0.005; F_[5]_ = 28.98, *p* < 0.001; F_[5]_ = 17.70, *p* = 0.003; F_[2]_ = 3.46, *p* = 0.040; F_[2]_ = 3.79, *p* = 0.031; respectively).

The post hoc analysis revealed that ASD adolescents were characterized by significantly higher levels of IL-1α, IL-1β, IL-2, IL-4, IL-6, IL-8, IL-10, M1 profile, and anti-inflammatory profile compared to the control group (*p* = 0.025; *p* = 0.005; *p* = 0.043; *p* = 0.021; *p* < 0.001; *p* = 0.010; *p* < 0.001; *p* = 0.011; *p* = 0.002; respectively). The post hoc analysis revealed no significant differences between the ASD females and control females. The post hoc analysis revealed that ASD males were characterized by significantly higher levels of IL-6, IL-10, and anti-inflammatory profile and a significantly lower Th1/Th2+Treg ratio compared to the control males (*p* = 0.021; *p* = 0.028; *p* = 0.032; *p* = 0.038; respectively). The post hoc analysis revealed that ADHD adolescents were characterized by significantly higher levels of IL-1β, IL-6, TNF-α, M1 profile, proinflammatory profile, and proinflammatory/anti-inflammatory cytokine ratio compared to the control group (*p* < 0.001; *p* < 0.001; *p* < 0.001; *p* < 0.001; *p* = 0.004; *p* < 0.001; respectively). The post hoc analysis revealed that the ADHD females were characterized by significantly higher levels of TNF-α and proinflammatory/anti-inflammatory ratio compared to the control females (*p* = 0.001; *p* = 0.012; respectively). The post hoc analysis revealed that the ADHD males were characterized by significantly higher levels of IL-1β, IL-6, TNF-α, and M1 profile compared to the control males (*p* = 0.034; *p* < 0.001; *p* = 0.004; *p* < 0.001; respectively). The post hoc analysis revealed that the ASD adolescents were characterized by significantly higher levels of IL-1α, IL-2, IL-4, IL-8, IL-10, and anti-inflammatory profile, and significantly lower levels of TNF-α, M1 profile, and Th1/Th2+Treg ratio compared to the ADHD adolescents (*p* = 0.010; *p* < 0.001; *p* < 0.001; *p* = 0.002; *p* = 0.007; *p* < 0.001; *p* < 0.001; *p* = 0.011; *p* < 0.001; respectively). The post hoc analysis revealed no significant differences between the ASD females and ADHD females. The post hoc analysis revealed that the ASD males were characterized by significantly higher levels of IL-2 and IL-8 and significantly lower levels of TNF-α and Th1/Th2+Treg ratio compared to the ADHD males (*p* = 0.002; *p* = 0.048; *p* = 0.005; *p* = 0.032; respectively). No significant between-group and between-sex differences were found in the remaining parameters. No significant between-sex difference within individual groups was found in all the evaluated parameters. All the results are summarized in Table 3 and Table 4.

### 2.3. ROC Curve Analyses

The ROC curve analyses were performed to assess the predictive performance of significantly changed parameters for ASD as well as ADHD diagnoses. In addition, as the combination of different parameters increases their sensitivity as well as specificity as a diagnostic tool [81], we performed a ROC analysis of the combination of all significantly changed parameters between the ASD adolescents and controls, as well as ADHD adolescents and controls. To be able to combine these parameters, standardized data according to the z formula were used.

#### 2.3.1. ROC Curve Analysis for Whole ASD Group

**Figure 2:**
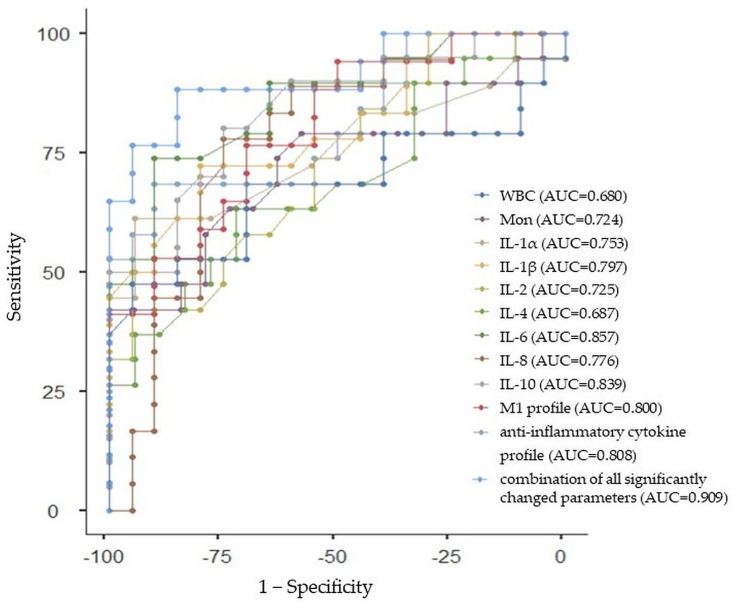
Receiver-operating characteristic curve analysis for the prediction of autism spectrum disorder (ASD) using white blood cells (WBC), monocytes (Mon), interleukin (IL)-1α, IL-1β, IL-2, IL-4, IL-6, IL-8, and IL-10, macrophages (M)1 profile, anti-inflammatory cytokine profile, and the combination of all significantly changed parameters between ASD and control group. AUC—area under the curve.

All of the individual parameters that differed in a significant way between ASD and the control group had good prediction abilities for ASD, with an AUC greater than 0.7, specifically for monocytes (AUC: 0.724, 95%CI: 0.566, 0.882), IL-1α (AUC: 0.753, 95%CI: 0.601, 0.905), IL-1β (AUC: 0.797, 95%CI: 0.657, 0.937), IL-2 (AUC: 0.725, 95%CI: 0.567, 0.883), IL-6 (AUC: 0.857, 95%CI: 0.737, 0.977), IL-8 (AUC: 0.776, 95%CI: 0.630, 0.922), IL-10 (AUC: 0.839, 95%CI: 0.713, 0.965), M1 profile (AUC: 0.800, 95%CI: 0.661, 0.939), and anti-inflammatory cytokine profile (AUC: 0.808, 95%CI: 0.671, 0.945), except for WBCs and IL-4, with an AUC under 0.7 (AUC: 0.680, 95%CI: 0.513, 0.847; AUC: 0.687, 95%CI: 0.521, 0.853, respectively). The diagnostic ability of ASD was improved by the combination of all significantly changed parameters with a sensitivity of 88.24% and specificity of 85.00% (AUC = 0.909, 95% Cl: 0.813, 1.005). The ROC curve analysis with the AUC, sensitivity, and specificity estimations is summarized in Table 5 and Figure 2.

#### 2.3.2. ROC Curve Analysis for ASD Females

As there were no significantly changed parameters between the ASD females and control females, the ROC analysis was not performed.

#### 2.3.3. ROC Curve Analysis for ASD Males

**Figure 3:**
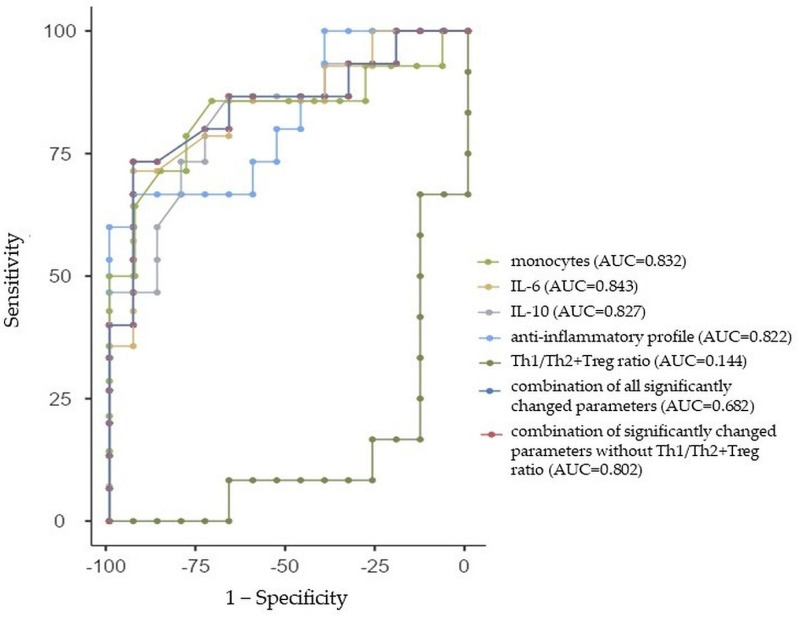
Receiver-operating characteristic curve analysis for the prediction of autism spectrum disorder (ASD) in males using monocytes (Mon), interleukin (IL)-6 and IL-10, anti-inflammatory cytokine profile, Th1/Th2+Treg ratio, the combination of all significantly changed parameters, and the combination of significantly changed parameters without Th1/Th2+Treg ratio between ASD males and control males. AUC—area under the curve.

All of the individual parameters that differed in a significant way between ASD males and the control males had good prediction abilities for ASD, with an AUC greater than 0.8, specifically monocytes (AUC: 0.832, 95%CI: 0.682, 0.982), IL-6 (AUC: 0.843, 95%CI: 0.698, 0.988), IL-10 (AUC: 0.827, 95%CI: 0.675, 0.979), and the anti-inflammatory profile (AUC: 0.822, 95%CI: 0.669, 0.975), except for the Th1/Th2+Treg ratio with an AUC of 0.144 (95%CI: 0.005, 0.283). Similarly, we performed the ROC analysis for the combination of all significantly changed parameters between adolescent ASD males and control males with a sensitivity of 68.06% and specificity of 64% (AUC = 0.682, 95%Cl: 0.489, 0.875). As the Th1/Th2+Treg ratio had too low of an AUC, we further performed a ROC analysis of significantly changed parameters without this ratio with a sensitivity of 75% and specificity of 76.67% (AUC = 0.802, 95%Cl: 0.641, 0.963). The ROC curve analysis with the AUC, sensitivity, and specificity estimations is summarized in Table 6 and Figure 3.

#### 2.3.4. ROC Curve Analysis for Whole ADHD Group

**Figure 4:**
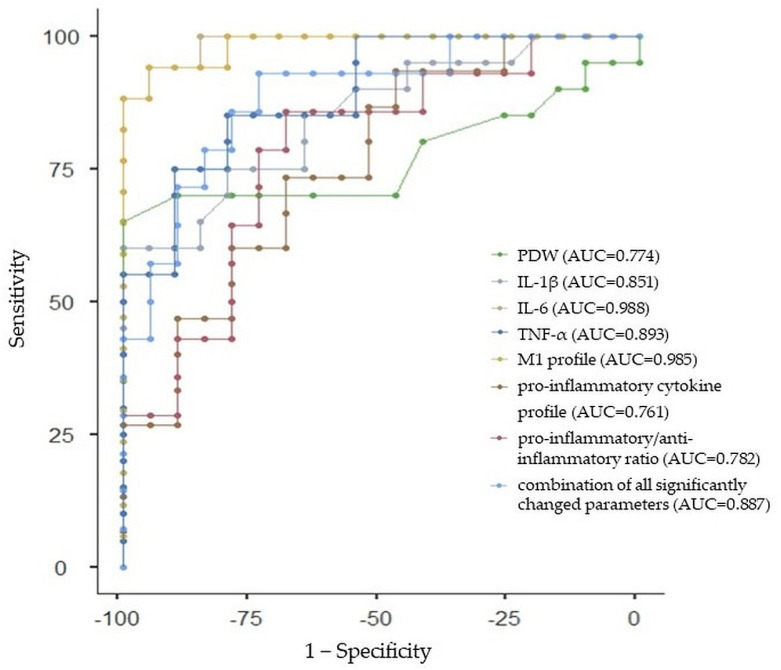
Receiver-operating characteristic curve analysis for the prediction of attention deficit/hyperactivity disorder (ADHD) using platelet distribution width (PDW), interleukin (IL)-1β and IL-6, tumor necrosis factor-alpha (TNF-α), macrophages (M)1 profile, proinflammatory cytokine profile, proinflammatory/anti-inflammatory ratio, and the combination of all significantly changed parameters between ADHD and control group. AUC—area under the curve.

All of the individual parameters that differed in a significant way between ADHD and the control group had good prediction abilities for ADHD, with an AUC greater than 0.7, specifically PDW (AUC: 0.774, 95%CI: 0.627, 0.921), IL-1β (AUC: 0.851, 95%CI: 0.729, 0.973), IL-6 (AUC: 0.988, 95%CI: 0.953, 1.023), TNF-α (AUC: 0.893, 95%CI: 0.789, 0.997), M1 profile (AUC: 0.985, 95%CI: 0.946, 1.024), proinflammatory cytokine profile (AUC: 0.761, 95%CI: 0.611, 0.911), and proinflammatory/anti-inflammatory ratio (AUC: 0.782, 95%CI: 0.638, 0.926). Similarly, we performed the ROC analysis of the combination of all significantly changed parameters between adolescent ADHD and control participants with a sensitivity of 92.86% and specificity of 73.68% (AUC = 0.887, 95%Cl: 0.780, 0.994). The ROC curve analysis with AUC, sensitivity, and specificity estimations is summarized in Table 7 and Figure 4.

#### 2.3.5. ROC Curve Analysis for ADHD Females

**Figure 5:**
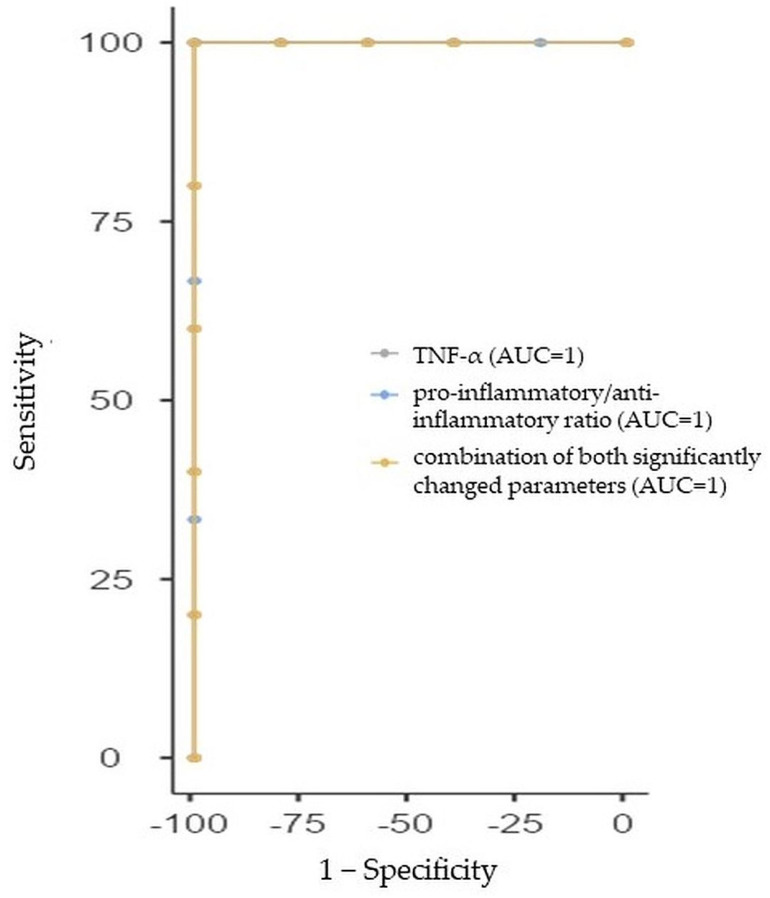
Receiver-operating characteristic curve analysis for the prediction of attention deficit/hyperactivity disorder (ADHD) in females using tumor necrosis factor-alpha (TNF-α), proinflammatory cytokine profile, and the combination of both significantly changed parameters between ADHD females and control females. AUC—area under the curve.

Both parameters (TNF-α and proinflammatory/anti-inflammatory ratio) that differed in a significant way between ADHD females and the control females had excellent prediction abilities for ADHD in females with AUC = 1 (95%CI: 1, 1, for both). Similarly, we performed the ROC analysis of the combination of these two significantly changed parameters between adolescent ADHD females and control females with a sensitivity of 100% and specificity of 100% (AUC = 1, 95%Cl: 1, 1). The ROC curve analysis with AUC, sensitivity, and specificity estimations is summarized in Table 8 and Figure 5.

#### 2.3.6. ROC Curve Analysis for ADHD Males

**Figure 6:**
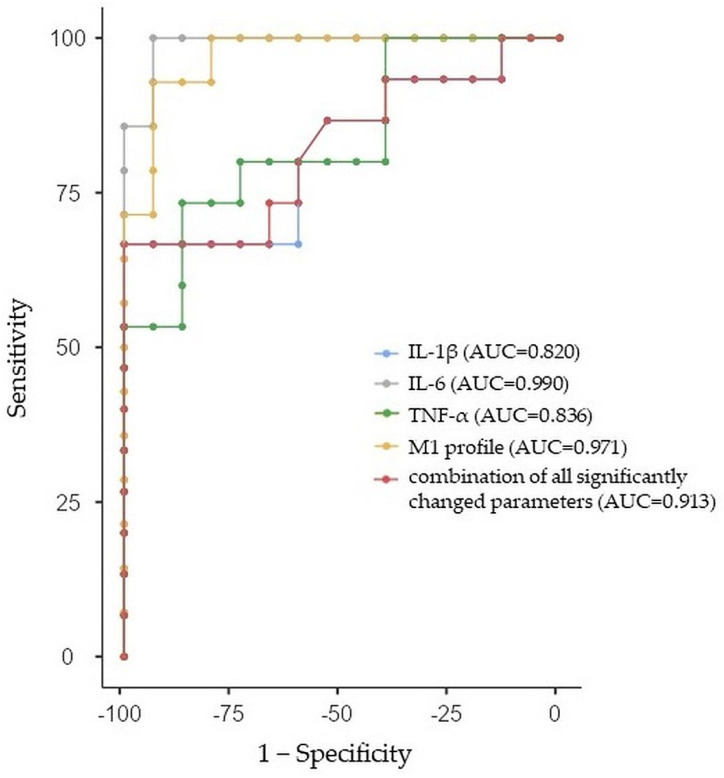
Receiver-operating characteristic curve analysis for the prediction of attention deficit/hyperactivity disorder (ADHD) in males using interleukin (IL)-1β and IL-6, tumor necrosis factor-alpha (TNF-α), and the combination of all significantly changed parameters between ADHD males and control males. AUC—area under the curve.

All of the individual parameters that differed in a significant way between ADHD males and the control males had good prediction abilities for ADHD in females with an AUC greater than 0.8, specifically IL-1β (AUC: 0.820, 95%CI: 0.666, 0.974), IL-6 (AUC: 0.990, 95%CI: 0.953, 1.027), TNF-α (AUC: 0.836, 95%CI: 0.688, 0.984), and M1 profile (AUC: 0.971, 95%CI: 0.908, 1.034). Similarly, we performed a ROC analysis of the combination of all significantly changed parameters between adolescent ADHD males and control males with a sensitivity of 83.05% and specificity of 83.33% (AUC = 0.913, 95%Cl: 0.804, 1.022). The ROC curve analysis with AUC, sensitivity, and specificity estimations is summarized in Table 9 and Figure 6.

## 3. Discussion

The main significant findings of this study could be summarized as follows: (1) increased levels of WBCs, monocytes, IL-1α, IL-1β, IL-2, IL-6, IL-8, and M1 profile, indicating the activation of the inflammatory response system in adolescent ASD; (2) increased levels of IL-4, IL-10, and anti-inflammatory profile, indicating the activation of the compensatory immune response system reflecting the potential counter-regulatory loop in adolescent ASD, predominantly emphasized in adolescent ASD males; (3) increased levels of IL-1β, IL-6, TNF-α, M1 profile, proinflammatory profile, and the proinflammatory/anti-inflammatory cytokine ratio, indicating the predominant activation of the inflammatory response system in adolescent ADHD of both sexes; however, it seemed to be more pronounced in adolescent ADHD males than females; (4) increased levels of IL-1α, IL-2, IL-4, IL-8, IL-10, and anti-inflammatory profile in association with decreased levels of TNF-α, M1 profile, and Th1/Treg+Th2 ratio in ASD adolescents compared to ADHD ones, indicating that immune dysregulation seemed to differ in these two neurodevelopmental disorders, particularly in males; (5) good ability of almost all significantly changed parameters for ASD and ADHD prediction with respect to sex assessed with a ROC analysis with an AUC estimation.

Systemic inflammation has been proposed to play an important role in psychiatric disorders including ASD and ADHD [82]; however, the relationships between these neurodevelopmental disorders and systemic inflammation remain elusive. Regarding the evaluation of CBC-derived markers and ratios representing one easy way to determine the presence of inflammation, our study revealed significantly increased levels of WBCs and monocytes in ASD adolescents compared to controls. In this context, WBCs as overall immune system activity markers include different types of cells, namely, neutrophils (whose increased levels have been predominantly associated with acute inflammation), lymphocytes and monocytes (whose increased levels have been mainly associated with chronic inflammation), eosinophils, and basophils [35,83,84]. In this way, our findings indicated a chronically activated inflammatory response system in ASD adolescents, particularly in ASD adolescent males, and were partially in accordance with a recent meta-analysis reporting increased blood levels of WBCs, neutrophils, and monocytes in ASD patients (age range: 3–22) [35]. With respect to ADHD, our study revealed increased PDW in adolescent ADHD compared to controls. PDW as a common platelet biomarker measuring platelet size variability indicates the heterogeneity in the morphology of platelets [85,86]. There is a mutual relationship between platelets and inflammation. While platelets can participate in inflammatory processes through the release of small molecules and proteins [87], numerous proinflammatory mediators, such as IL-1, released during inflammation can contribute to platelet activation. Consequently, the platelet activation can cause changes in the shape and formation of pseudopodia, resulting in larger PDW values [88,89]. Our result showing increased PDW in ADHD adolescents was in accordance with a recent study evaluating hematological inflammatory markers in ADHD children and adolescents [36], and could reflect an inflammatory condition as a platelet marker in adolescent ADHD.

From the proinflammatory cytokine perspective, our study revealed significantly increased levels of proinflammatory IL-1α, IL-1β, IL-2, IL-6, and IL-8 in association with an increased M1 profile in ASD adolescents and significantly higher levels of IL-1β, IL-6, and TNF-α in association with an increased M1 profile, proinflammatory profile, and proinflammatory/anti-inflammatory ratio in ADHD adolescents compared to controls. Through complex cytokine networks, alterations in the peripheral cytokine production can modulate the development and physiology of the CNS via influencing neural plasticity, neurotransmitter function, and neuroendocrine activity. More specifically, IL-1β represents a major proinflammatory cytokine, playing a crucial role in inflammatory responses. Moreover, IL-1β contributes to the increased expression of adhesion molecules, which, consequently, can support the infiltration of tissue by inflammatory cells from the circulation, resulting in chronic inflammation [90,91]. In the brain, IL-1β can exert a myriad of effects, such as neuronal proliferation, differentiation, and apoptosis [92]. Additionally, IL-1β enhances the production of another proinflammatory cytokine, IL-6, secreted by various cells, including lymphocytes, macrophages, neurons, microglia, and astrocytes. Within the CNS, IL-6 plays a key role in the development and functioning of the brain by influencing neurogenesis, neuronal differentiation, myelination, and synapse formation [59,93,94,95]. Moreover, IL-6 acts as a facilitator of the CNS–immune interplay [96]. In this way, the dysregulation of IL-1β and IL-6 (altered in ASD and ADHD adolescents in this study) could represent potential biomarkers of both neurodevelopmental disorders, ASD and ADHD. Another proinflammatory cytokine, TNF-α (similarly to IL-1β and IL-6), can cross into the brain from peripheral blood and directly modulate CNS functioning [97]. Our study revealed the elevation of TNF-α in ADHD adolescents compared to controls, which was contrary to the previous findings of unchanged [61] or even decreased levels of TNF-α [64] in ADHD. As a TNF-α imbalance (too much or too little) has been shown to impair cognitive functions [98,99] and monoamine turnover [100], increased TNF-α levels in ADHD adolescents could be linked to impaired ADHD-related cognitive performance. Interestingly, while in the ADHD group enhanced peripheral proinflammatory activity was found within both sexes, although showing to be more pronounced in males, compared to control subjects (i.e., increased levels of TNF-α and proinflammatory/anti-inflammatory ratio in adolescent ADHD females compared to control females and increased levels of IL-1β, IL-6, TNF-α, and M1 profile in adolescent ADHD males compared to control males), in the ASD group, significantly increased proinflammatory cytokine IL-6 was revealed only in adolescent ASD males when compared to control males. These findings support the suggestion that different mechanisms may underlie ASD and ADHD in males and females, with males possibly being more vulnerable to immune-linked risk factors for neurodevelopmental disorders [74].

There are several mechanisms through which neuroinflammation can alter the development of the brain and, thus, lead to neurodevelopmental disorders, the including activation of glia, increased oxidative stress, the abnormal development of neurons, and disturbed function of neurotransmitters [13,101,102,103,104]. Moreover, the neuroinflammation driven by proinflammatory cytokines can originate from different pathways: (1) proinflammatory cytokines, which arise from maternal inflammation during pregnancy crossing the placenta into the fetal circulation and, consequently, altering the neurodevelopment after crossing the BBB of the fetus [105,106]; and/or (2) an excessive release of peripheral proinflammatory cytokines passing through the BBB into the brain, consequently, altering brain development through the stimulation of aberrant neurogenesis and modulating the formation of synapses, and neuronal and neurotransmitter functions [10]. Neuroinflammation can further promote the disruption of BBB integrity, allowing for the free entrance of other proinflammatory molecules into the brain, further modulating neural function. Thus, neuroinflammation can represent one of the principal mechanisms in ASD and ADHD pathophysiology [13,107]; however, the question of whether immune dysregulation in ASD and ADHD is a primary cause or the consequence remains open.

A better understanding of the inflammatory role in ASD and ADHD pathophysiologies could bring novel insights into these diseases´ therapeutic strategies. More specifically, a growing body of studies examined the effectiveness of medications with primary or additional anti-inflammatory properties in ASD, including antiviral medications such as amantadine (e.g., [108]), corticosteroids such as prednisolone (e.g., [109]), antibiotics such as minocycline (e.g., [110], the selective inhibitor of cyclooxygenase-2 celecoxib (e.g., [111]), noncompetitive N-methyl-D-aspartate receptor antagonist memantine [112], and flavonoids such as luteolin and quercetin (e.g., [113]) and others (for a review, see, e.g., [114,115,116,117]). Contrary to ASD, less evidence of targeting inflammation in ADHD has been presented thus far. In this context, antioxidants, such as N-acetylcysteine, sulforaphane, and omega-3 fatty acids, have been proposed for potential application in multitarget adjuvant therapy in ADHD [118]. Furthermore, the efficacy of noncompetitive N-methyl-D-aspartate receptor antagonist memantine monotherapy in ADHD has been described [112]. Currently, the potential of anticytokine therapy via cytokine antibodies/inhibitors allowing for the precise targeting of specific immune pathways has been extensively discussed [119]. Thus, our findings of increased proinflammatory cytokine levels in ASD (i.e., IL-1α, IL-1β, IL-2, IL-6, and IL-8) and ADHD (i.e., IL-1β, IL-6, and TNF-α) adolescents with respect to sex could offer a way of achieving targeted therapeutic interventions that could contribute to personalized medicine in both neurodevelopmental disorders.

On the other side of immune responses, a compensatory immune system activation mediating a counter-regulating effect arises from the differentiation of naïve T helper (Th0) cells into Treg and/or Th2 cells, releasing anti-inflammatory cytokines such as IL-10 and IL-4 [67]. IL-10 represents the most frequently studied anti-inflammatory cytokine, playing a crucial role in the modulation of immune responses through the regulation of Th1 cells, monocytes, and macrophages activity, and the suppression of proinflammatory cytokines such as IL-1β, IL-6, IFN-γ, and TNF-α release [46,120,121,122]. IL-4, as another important anti-inflammatory cytokine, mediates the M2 macrophage activation and, through the release of TGF-β, IL-1RA, and IL-10, diminishes enhanced proinflammatory activity [123]. Given the potential of IL-10 and IL-4 to modulate brain inflammatory conditions [46,124], further research is needed to reveal the precise regulatory mechanisms of these cytokines’ production within the CNS and their impact on neuroinflammatory responses. With respect to IL-10 in ASD, studies reported inconsistent findings. The majority of studies revealed unchanged levels of IL-10 in ASD patients compared to controls [47,125,126,127], but some studies reported decreased levels of IL-10 [68,128,129] in ASD patients compared to controls. Contrarily, our study revealed increased plasma IL-10 levels in ASD adolescents, predominantly in ASD males. In this context, it is important to note that during the onset of adolescence, there is an approximately 30-fold increase in the production of testosterone in boys [130], which has been shown to modulate inflammation through an increase in IL-10 production [131,132]. Moreover, ASD individuals during early adolescence have been reported to have significantly higher levels of testosterone compared to age-matched typically developing peers, indicating a unique role of testosterone in ASD, especially during the periods associated with dynamic changes in hormones [133]. Thus, we suggest that increased levels of anti-inflammatory IL-10 in this study could have resulted from a testosterone elevation either due to the puberty-linked adolescent age period and/or ASD diagnosis. With respect to IL-4 in ASD, our study revealed increased levels of this cytokine in ASD adolescents, which was consistent with several recent studies, including the recent meta-analysis [134,135,136]. Based on our findings, increased anti-inflammatory activity could indicate a potential feedback loop on increased proinflammatory activity in adolescent ASD patients, particularly in males.

Lastly, there is still a lack of objective biomarkers that could be used as indicators for ASD and ADHD diagnoses and prognoses, as well as the risk assessment of the diseases. In this study, the ROC analysis revealed that almost all significantly changed parameters between ASD and the control group (i.e., monocytes, IL-1α, IL-1β, IL-2, IL-6, IL-8, IL-10, the M1 profile, and anti-inflammatory profile) indicated good prediction abilities for ASD (i.e., AUC was greater than 0.7), except for WBCs, with AUC = 0.680 and IL-4 with AUC = 0.687. Moreover, the diagnostic ability of adolescent ASD was improved through the combination of all parameters, which significantly differed between ASD and the control group (AUC = 0.909, indicating excellent predictive performance). Further, while the ROC analysis has not been performed in ASD females, according to the lack of significantly changed parameters between ASD females and control females, the ROC analysis in ASD males revealed that almost all significantly changed parameters (i.e., monocytes, IL-6, IL-10, and the anti-inflammatory cytokine profile) between ASD males and control males indicated good prediction abilities (AUC greater than 0.8), except for the Th1/Th2+Treg ratio (AUC = 0.144) for ASD in males. While the AUC for the combination of all parameters that significantly differed between ASD males and the control males was 0.682, the AUC for the combination of parameters that significantly differed between ASD males and the control males without the Th1/Th2+Treg ratio was 0.802. With respect to ADHD, the ROC analysis revealed that all significantly changed parameters between ADHD and the control group (i.e., PDW, IL-1β, IL-6, TNF-α, the M1 profile, proinflammatory profile, and proinflammatory/anti-inflammatory ratio) indicated good prediction abilities for ADHD (i.e., AUC was greater than 0.7). The AUC for the combination of all parameters that significantly differed between ADHD and the control group was 0.887. The ROC analysis in ADHD males revealed that significantly changed parameters (i.e., IL-1β, IL-6, TNF-α, and the M1 profile) and their combination indicated good prediction abilities (AUC greater than 0.8) for ADHD in males, while the ROC analysis in ADHD females revealed that significantly changed parameters (i.e., TNF-α and proinflammatory/anti-inflammatory ratio) and their combination between ADHD females and control females indicated excellent prediction abilities (AUC = 1) for ADHD in females. To the best of our knowledge, this was the first study using combined ROC curve analyses with respect to sex to assess the diagnostic ability of CBC parameters and cytokines in adolescent ASD and ADHD.

The limitation of this study was its relatively small group of ASD and ADHD adolescents; thus, the findings cannot be extrapolated to the general population. Future research should focus on larger patient groups with respect to sex differences and also younger age periods for the detection of an immune system developmental trajectory. Moreover, this work represents a cross-sectional study; thus, the changes in CBC parameters and cytokines of each participant over time were unknown. In this context, future studies could implement repeated CBC and cytokine measurements in ASD, ADHD, and control participants to determine the developmental progression.

## 4. Materials and Methods

The study was conducted according to the guidelines of the Declaration of Helsinki and approved by the ethics committee of the Jessenius Faculty of Medicine in Martin, Comenius University in Bratislava (protocol codes EK2058/2017 and EK88/2019). All adolescents and their guardians were carefully informed about the study protocol and informed consent was obtained from all subjects/guardians involved in the study.

### 4.1. Subjects

**Figure 7:**
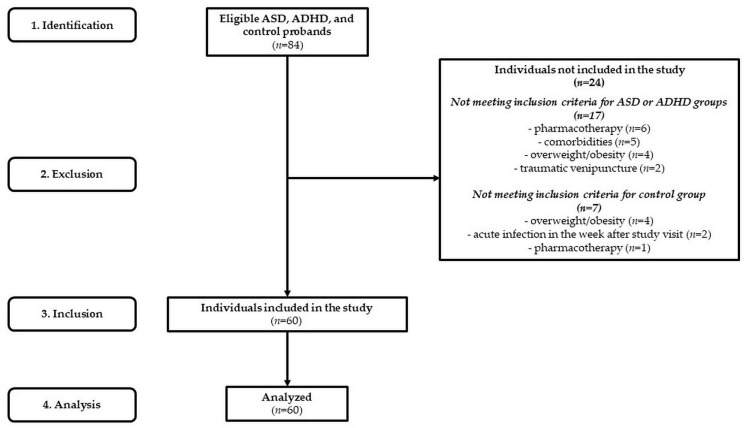
STROBE flow chart of participants. ASD—autism spectrum disorder; ADHD—attention deficit/hyperactivity disorder.

We examined a sample of 60 Caucasian adolescents (Figure 7), of which (1) 20 drug-naïve ASD adolescents were recruited from the regional Autism Centre and inpatients admitted to the Psychiatric Clinic of Jessenius Faculty of Medicine and University Hospital in Martin (mean age: 12.4 ± 1.9 years and BMI: 19.7 ± 2.6 kg/m^2^; 5 females with mean age: 11.6 ± 0.7 years and BMI: 21.3 ± 1.7 kg/m^2^; 15 males with mean age: 12.7 ± 0.5 years and BMI: 19.2 ± 0.5 kg/m^2^), (2) 20 drug-naïve ADHD adolescents were recruited from inpatients admitted to the Psychiatric Clinic of Jessenius Faculty of Medicine and University Hospital in Martin (mean age: 13.4 ± 1.8 years and BMI: 19.9 ± 2.6 kg/m^2^; 5 females with mean age: 13.0 ± 1.1 years and BMI: 18.0 ± 0.7 kg/m^2^; 15 males with mean age: 13.5 ± 0.4 years and BMI: 20.6 ± 0.7 kg/m^2^), and (3) 20 were age- and gender-matched controls (mean age: 13.2 ± 1.9 years and BMI: 19.5 ± 2.5 kg/m^2^; 5 females with mean age: 14.2 ± 0.9 years and BMI: 20.2 ± 1.3 kg/m^2^; 15 males with mean age: 12.8 ± 0.5 years and BMI: 19.2 ± 0.6 kg/m^2^).

#### 4.1.1. ASD Diagnosis

The diagnosis of ASD was confirmed by child and adolescent psychiatric specialists according to the Diagnostic and Statistical Manual of Mental Disorders, DSM-5 [4]. Moreover, intellectual functioning through the Wechsler Intelligence Scale for Children was evaluated by a licensed clinical psychologist. The inclusion criteria for the ASD group were the following: psychiatric examination by a child psychiatrist for ASD diagnosis without comorbidities, IQ above 70, adolescent age (10–19 years), and no pharmacotherapy.

#### 4.1.2. ADHD Diagnosis

The diagnosis of ADHD was assessed via clinical investigations by child and adolescent psychiatric specialists according to the DSM-5 [4]. The inclusion criteria for the ADHD group were the following: psychiatric examination by a child psychiatrist for ADHD diagnosis without comorbidities, adolescent age (10–19 years), and no pharmacotherapy.

#### 4.1.3. Exclusion Criteria

The following exclusion criteria for all subjects were applied: overweight/obesity, allergies, acute/chronic infections, endocrine diseases, immune diseases, systemic diseases, cancer, and pharmacotherapy. The possibility of participants´ allergies, acute/chronic infections, endocrine diseases, immune diseases, systemic diseases, cancers, and pharmacotherapies was excluded through the participants´ and their guardians´ self-report and the careful inspection of participants´ medical records. Control subjects never suffered from a mental disorder. All participants had to meet the condition of atraumatic venous blood sampling. Moreover, guardians had to notify the investigator if their adolescent developed the symptoms of an acute infection in one week after they participated in the study.

### 4.2. Blood Analysis

We collected 5 mL of fasting peripheral venous blood samples in EDTA test tubes. An automated hematology analyzer (Mindray BC-5500, Guangdong, China) was used to analyze whole blood specimens for the assessment of levels of WBCs, neutrophils, monocytes, lymphocytes, platelets, MPV, and PDW, with a subsequent estimation of selected ratios (NLR, PLR, LMR, MLR, PMR, MPVLR, and MPVPR). To be able to estimate the ratios from raw data in different units, standardized data according to the z formula were used: z = (x − M)/SD [137], where z = standardized score; x = proband´s raw data; M = mean level of the parameter in the combined group (i.e., ASD, ADHD, and control groups together); SD = standard deviation of the parameter in the combined group. Subsequently, we centrifuged the blood samples for 15 min at 2500 rpm and 4 °C (refrigerated centrifugation, Hettich Universal 320R, Tuttlingen, Germany), and the obtained plasma was stored at −80 °C until the analysis. We analyzed and quantified several cytokines, including IFN-γ, IL-1α, IL-1β, IL-2, IL-4, IL-6, IL-8, IL-10, and TNF-α, using biochip array technology (Evidence Investigator, Randox, Crumlin, County Antrim, Northern Ireland, UK). Randox provided plasma assays in a separate kit, which contained biochip cartridges, calibrators, an assay diluent, conjugate, wash buffer, and signal reagent. For each assay series, we performed a nine-point calibration, and quality controls were used for running the validation. Cytokines present in the proband´s sample bound to the specific ligands of the biochip, and the degree of binding was evaluated with a charge-coupled device camera and imaging system. Consequently, raw cytokine data (in a logarithmic transformation, according to [138]) were transformed into standardized z scores, which were then used to assess the following: several cytokine profiles, namely, Th1 profile = zIL-2 + zIFN-γ; M1 profile = zIL-1β + zIL-6 + zTNF-α; proinflammatory cytokine profile = zIL-1α + zIL-1β + zIL-2 + zIL-6 + zIL-8 + zIFN-γ + zTNF-α; anti-inflammatory cytokine profile = zIL-4 + zIL-10; and cytokine ratios, namely, Th1/Th2 ratio = z(zIFN-γ + zIL-2) − zIL-4; Th1/Treg ratio = z(zIFN-γ + zIL-2) − zIL-10; Th1/Th2+Treg ratio = z(zIFN-γ + zIL-2) − z(zIL-4 + zIL-10); proinflammatory/anti-inflammatory cytokine ratio = z(zIL-1α + zIL-1β + zIL-2 + zIL-6 + zIL-8 + zIFN-γ + zTNF-α) – z(zIL-4 + zIL-10)—a subtraction was performed in the ratios because these values were logarithmically transformed. A similar assessment of the individual cytokines, cytokine profiles, and ratios was used in our previous study, which focused on adolescent depression [139].

### 4.3. Statistical Analysis

The data were explored and analyzed in jamovi version 1.2.27 (Sydney, Australia). Data distributions (Gaussian/non-Gaussian) were evaluated using the Shapiro–Wilk normality test. The between-group, between-sex, and mixed-group × sex comparisons were performed using an analysis of variance (ANOVA) and Bonferroni post hoc test for evaluated data with normal distributions and using the Kruskal–Wallis test with the Dwass–Steel–Critchlow–Fligner post hoc test for evaluated data with non-normal distributions. Results of the test with a *p*-value below 0.05 were considered statistically significant. Data were expressed as mean ± SD or median (interquartile range). Effect size estimations through the use of Cohen’s d were applied. In addition, a ROC curve analysis was performed to determine the AUCs, sensitivities, and specificities of the identified parameters for predicting ASD and ADHD. A ROC curve is a graphical display plotting sensitivity estimates (probability of a true positive) against one minus specificity (probability of a false positive) of a biomarker for all possible threshold values. The optimal cut-off points for individual parameters were determined using the maximum value of Youden´s index. The performance of a biomarker was evaluated using the AUC, with an AUC value close to 1.00 indicating an excellent predictive marker, while a biomarker with an AUC close to 0.50 reflected no diagnostic efficacy. Moreover, an AUC close to 1.00 was associated with a sufficient biomarker sensitivity and specificity recorded through the ROC curve analysis [140].

## 5. Conclusions

This study revealed increased levels of WBC, monocytes, IL-1α, IL-1β, IL-2, IL-4, IL-6, IL-8, IL-10, M1 profile, and anti-inflammatory profile, indicating heightened proinflammatory as well as anti-inflammatory activity in ASD adolescents, and increased PDW, IL-1β, IL-6, TNF-α, M1 profile, proinflammatory profile, and proinflammatory/anti-inflammatory ratio indicating dominant proinflammatory activity in ADHD adolescents. With respect to sex, while ASD females showed no differences in all evaluated parameters compared to control females, ASD males showed increased monocytes, IL-6, IL-10, and anti-inflammatory profile and a decreased Th1/Th2+Treg ratio compared to the control males. Concerning ADHD, while ADHD females showed increased level of TNF-α and proinflammatory/anti-inflammatory ratio compared to the control females, ADHD males showed increased levels of IL-1β, IL-6, TNF-α, and M1 profile compared to the control males. Thus, immune dysregulation appeared to be different and more pronounced in males for both neurodevelopmental disorders at the adolescent age (see Table 10).

## Figures and Tables

**Table 1:** Evaluation of the complete blood count parameters and selected ratios between individual groups (i.e., ASD, ADHD, and control groups) with respect to sex.

Parameter	Controls ^a^ (*n* = 20)	Control Females ^b^ (*n* = 5)	Control Males ^c^ (*n* = 15)	ASD ^d^ (*n* = 20)	ASD Females ^e^ (*n* = 5)	ASD Males ^f^ (*n* = 15)	ADHD ^g^ (*n* = 20)	ADHD Females ^h^ (*n* = 5)	ADHD Males ^i^ (*n* = 15)
WBC (10^9^/L)	6.18 ± 0.97	6.15 ± 0.46	6.19 ± 0.26	7.37 ± 1.89	6.64 ± 0.72	7.63 ± 0.53	7.21 ± 1.42	7.02 ± 0.33	7.28 ± 0.43
Neu (10^9^/L)	2.78 ± 0.73	3.12 ± 0.40	2.66 ± 0.17	3.46 ± 1.19	3.13 ± 0.40	3.58 ± 0.34	3.50 ± 0.99	2.94 ± 0.41	3.66 ± 0.27
Lym (10^9^/L)	2.56 (2.20, 3.01)	2.21 (2.11, 2.45)	2.62 (2.40, 3.13)	2.63 (2.25, 3.59)	2.46 (2.27, 2.73)	2.65 (2.28, 3.59)	2.90 (1.91, 3.73)	3.28 (2.87, 4.36)	2.71 (1.90, 3.40)
Mon (10^9^/L)	0.47 (0.40, 0.53)	0.59 (0.50, 0.62)	0.46 (0.39, 0.48)	0.58 (0.49, 0.70)	0.50 (0.41, 0.61)	0.62 (0.50, 0.76)	0.52 (0.42, 0.68)	0.41 (0.38, 0.44)	0.56 (0.45, 0.72)
PLT (10^9^/L)	277 (241, 297)	277 (276, 300)	266 (241, 295)	313 (255, 345)	331 (297, 365)	296 (251, 344)	302 (263, 348)	288 (265, 331)	303 (274, 352)
MPV (fL)	8.55 ± 0.71	8.66 ± 0.43	8.52 ± 0.17	8.34 ± 0.81	7.68 ± 0.33	8.58 ± 0.19	8.61 ± 0.68	8.66 ± 0.32	8.59 ± 0.18
PDW (%)	16.60 ± 0.38	16.70 ± 0.10	16.60 ± 0.11	17.00 ± 0.83	16.90 ± 0.52	17.00 ± 0.20	17.30 ± 0.87	17.10 ± 0.47	17.30 ± 0.22
NLR	0.97 (0.80, 1.37)	1.39 (1.36, 1.41)	0.91 (0.80, 1.10)	1.15 (1.02, 1.43)	1.07 (0.98, 1.23)	1.15 (1.07, 1.45)	1.21 (0.91, 1.73)	0.75 (0.57, 1.03)	1.30 (0.98, 1.84)
PLR	103.0 (85.4, 113.0)	114.0 (113.0, 136.0)	101.0 (83.1, 104.0)	113.0 (99.7, 119.0)	96.3 (85.9, 129.0)	114.0 (110.0, 119.0)	102.0 (72.1, 125.0)	70.0 (66.1, 101.0)	115.0 (84.8, 140.0)
LMR	5.23 (4.56, 6.45)	4.21 (3.58, 4.42)	5.71 (4.88, 6.67)	4.92 (3.64, 6.52)	5.54 (4.92, 6.06)	4.30 (3.34, 6.59)	5.22 (4.17, 7.05)	8.55 (7.00, 8.63)	4.96 (3.91, 5.96)
MLR	0.19 (0.16, 0.22)	0.24 (0.23, 0.28)	0.18 (0.15, 0.21)	0.20 (0.15, 0.28)	0.18 (0.17, 0.20)	0.23 (0.15, 0.30)	0.19 (0.14, 0.24)	0.12 (0.12, 0.14)	0.20 (0.17, 0.26)
PMR	531 (466, 688)	476 (445, 600)	539 (493, 704)	409 (370, 590)	543 (454, 669)	408 (372, 579)	583 (415, 700)	602 (565, 846)	487 (404, 692)
MPVLR	−0.06 (−1.07, 2.82)	−1.01 (−1.23, −0.31)	0.25 (−0.58, 2.88)	−0.59 (−1.36, 0.86)	−0.59 (−2.57, 1.58)	−0.78 (−1.29, 0.66)	−0.23 (−0.59, 0.59)	−0.20 (−0.25, 0.58)	−0.29 (−1.41, 0.47)
MPVPR	−1.28 (−1.95, 0.19)	−1.26 (−5.68, −0.74)	−1.29 (−1.95, 0.20)	−0.84 (−1.45, −0.49)	−1.37 (−1.41, −1.13)	−0.78 (−1.69, −0.25)	−0.10 (−1.13, 0.61)	0.78 (0.28, 1.35)	−0.67 (−1.33, 0.57)

ASD—autism spectrum disorder; ADHD—attention deficit/hyperactivity disorder; WBC- white blood cells; Neu—neutrophils; Lym—lymphocytes; Mon—monocytes; PLT—platelets; MPV—mean platelet volume; PDW—platelet distribution width; NLR—neutrophils to lymphocytes ratio; PLR—platelets to lymphocytes ratio; LMR—lymphocytes to monocytes ratio; MLR—monocytes to lymphocytes ratio; PMR—platelets to monocytes ratio; MPVLR—MPV to lymphocytes ratio; MPVPR—MPV to platelets ratio. Data are expressed as mean ± SD or median (interquartile range). ^a^ indicates the control group; ^b^ indicates the control females; ^c^ indicates the control males; ^d^ indicates the ASD group; ^e^ indicates the ASD females; ^f^ indicates the ASD males; ^g^ indicates the ADHD group; ^h^ indicates the ADHD females; ^i^ indicates the ADHD males.

**Table 2:** Comparison of the complete blood count parameters and selected ratios between individual groups (i.e., ASD, ADHD, and control groups) with respect to sex.

Parameter	*p*-Value/ Cohen’s d a vs. d	*p*-Value/ Cohen’s d a vs. g	*p*-Value/ Cohen’s d d vs. g	*p*-Value/ Cohen’s d b vs. e	*p*-Value/ Cohen’s d b vs. h	*p*-Value/ Cohen’s d e vs. h	*p*-Value/ Cohen’s d c vs. f	*p*-Value/ Cohen’s d c vs. i	*p*-Value/ Cohen’s d f vs. i	*p*-Value/ Cohen’s d b vs. c	*p*-Value/ Cohen’s d e vs. f	*p*-Value/ Cohen’s d h vs. i
WBC (10^9^/L)	**0.042**/0.812	0.099/0.700	0.999/−0.112	0.999/0.332	0.999/0.585	0.999/0.253	0.175/0.971	0.818/0.731	0.999/−0.241	0.999/−0.027	0.999/−0.667	0.999/−0.174
Neu (10^9^/L)	0.104/0.694	0.082/0.737	0.999/0.042	0.999/0.012	0.999/−0.186	0.999/−0.198	0.231/0.931	0.126/1.019	0.999/0.087	0.999/0.466	0.999/−0.454	0.999/−0.740
Lym (10^9^/L)	0.785/0.321	0.793/0.393	0.998/0.071	0.861/0.536	0.621/1.076	0.936/0.540	0.999/0.245	0.999/0.159	0.994/−0.086	0.444/−0.485	0.999/−0.194	0.987/0.432
Mon (10^9^/L)	**0.047**/0.790	0.482/0.557	0.694/−0.233	0.998/−0.172	0.608/−0.737	0.960/−0.565	**0.033**/1.184	0.235/1.035	0.998/−0.149	0.429/0.625	0.895/−0.731	0.206/−1.146
PLT (10^9^/L)	0.180/0.741	0.064/0.556	0.999/−0.185	0.901/0.831	0.999/−0.160	0.991/−0.990	0.614/0.752	0.201/0.789	0.998/0.037	0.997/0.352	0.987/0.431	0.942/−0.597
MPV (fL)	0.999/−0.290	0.999/0.075	0.780/0.365	0.526/−1.368	0.999/0.000	0.526/1.368	0.999/0.082	0.999/0.102	0.999/0.021	0.999/0.120	0.293/−1.255	0.999/0.093
PDW (%)	0.319/0.540	**0.015**/0.938	0.678/0.398	0.999/0.268	0.999/0.589	0.999/0.321	0.999/0.624	0.118/1.029	0.999/0.405	0.999/0.182	0.999/−0.175	0.999/−0.259
NLR	0.416/0.275	0.600/0.446	0.995/0.171	0.936/−0.412	0.825/−1.017	0.825/−0.605	0.364/0.519	0.268/0.908	0.988/0.389	0.589/0.713	0.987/−0.218	0.462/−1.212
PLR	0.420/0.123	0.996/0.283	0.954/0.160	0.976/−0.348	0.352/−1.071	0.903/−0.723	0.229/0.296	0.758/0.747	0.999/0.451	0.083/0.736	0.999/0.093	0.589/−1.082
LMR	0.591/−0.333	0.979/−0.109	0.779/0.224	0.753/0.607	0.241/1.756	0.621/1.149	0.441/−0.705	0.445/−0.761	0.999/−0.055	0.154/−1.062	0.962/0.251	0.226/1.455
MLR	0.591/0.349	0.979/0.289	0.779/−0.060	0.753/−0.689	0.241/−1.297	0.621/−0.608	0.441/0.748	0.445/0.857	0.999/0.110	0.154/0.953	0.962/−0.483	0.226/−1.201
PMR	0.519/−0.241	0.989/−0.059	0.670/0.181	0.999/0.167	0.936/0.476	0.976/0.309	0.840/−0.358	0.970/−0.242	0.982/0.116	0.977/−0.339	0.999/0.186	0.977/0.379
MPVLR	0.763/−0.243	0.851/−0.067	0.877/0.176	0.999/0.029	0.621/1.206	0.903/1.176	0.902/−0.350	0.708/−0.495	0.999/−0.144	0.848/−0.433	0.999/−0.053	0.943/1.267
MPVPR	0.999/0.120	0.229/0.674	0.143/0.554	0.999/−0.374	0.684/0.394	0.276/0.768	0.999/−0.526	0.958/−1.105	0.943/−0.579	0.994/−0.899	0.916/−0.746	0.727/0.601

ASD—autism spectrum disorder; ADHD—attention deficit/hyperactivity disorder; WBC—white blood cells; Neu—neutrophils; Lym—lymphocytes; Mon—monocytes; PLT—platelets; MPV—mean platelet volume; PDW—platelet distribution width; NLR—neutrophils to lymphocytes ratio; PLR—platelets to lymphocytes ratio; LMR—lymphocytes to monocytes ratio; MLR—monocytes to lymphocytes ratio; PMR—platelets to monocytes ratio; MPVLR—MPV to lymphocytes ratio; MPVPR—MPV to platelets ratio. A value of *p* ≤ 0.05 (in bold) was considered statistically significant. a indicates the control group; b indicates the control females; c indicates the control males; d indicates the ASD group; e indicates the ASD females; f indicates the ASD males; g indicates the ADHD group; h indicates the ADHD females; i indicates the ADHD males.

**Table 3:** Evaluation **of** circulating cytokines, cytokine profiles, and cytokine ratios between individual groups (i.e., ASD, ADHD, and control groups) with respect to sex.

Parameter	Controls ^a^ (*n* = 20)	Control Females ^b^ (*n* = 5)	Control Males ^c^ (*n* = 15)	ASD ^d^ (*n* = 20)	ASD Females ^e^ (*n* = 5)	ASD Males ^f^ (*n* = 15)	ADHD ^g^ (*n* = 20)	ADHD Females ^h^ (*n* = 5)	ADHD Males ^i^ (*n* = 15)
Cytokines									
IL-1α (pg/mL)	0.21 (0.18, 0.22)	0.21 (0.19, 0.22)	0.21 (0.18, 0.22)	0.25 (0.21, 0.30)	0.70 (0.57, 0.74)	0.24 (0.21, 0.28)	0.13 (0.11, 0.21)	0.21 (0.08, 0.37)	0.13 (0.11, 0.17)
IL-1β (pg/mL)	0.88 (0.68, 0.97)	0.64 (0.62, 0.91)	0.88 (0.75, 1.05)	1.19 (0.93, 1.69)	1.08 (0.93, 2.06)	1.21 (0.97, 1.61)	1.28 (1.02, 2.83)	1.13 (1.11, 1.31)	1.80 (0.94, 2.97)
IL-2 (pg/mL)	1.68 (1.26, 2.21)	1.40 (1.01, 1.74)	1.88 (1.33, 2.38)	2.25 (1.70, 4.97)	5.12 (2.55, 7.66)	2.19 (1.70, 3.92)	1.19 (0.92, 1.68)	1.78 (1.28, 1.88)	1.07 (0.86, 1.28)
IL-4 (pg/mL)	1.89 ± 0.37	1.92 ± 0.13	1.88 ± 0.11	2.37 ± 0.72	2.61 ± 0.41	2.31 ± 0.18	1.69 ± 0.40	1.66 ± 0.15	1.70 ± 0.12
IL-6 (pg/mL)	0.52 (0.37, 0.63)	0.53 (0.38, 0.68)	0.52 (0.37, 0.61)	0.81 (0.66, 1.43)	1.35 (1.00, 1.85)	0.74 (0.65, 1.00)	1.17 (1.06, 1.45)	1.15 (1.10, 1.69)	1.21 (0.98, 1.43)
IL-8 (pg/mL)	3.38 (3.06, 3.88)	3.29 (3.19, 3.48)	3.70 (2.98, 4.36)	4.48 (3.90, 5.42)	5.85 (5.36, 6.29)	4.27 (3.83, 5.06)	3.05 (2.54, 3.44)	2.96 (2.67, 3.31)	3.08 (2.49, 3.46)
IL-10 (pg/mL)	0.61 (0.51, 0.70)	0.66 (0.52, 0.72)	0.58 (0.51, 0.67)	0.96 (0.70, 1.76)	1.77 (0.79, 1.99)	0.80 (0.68, 1.32)	0.54 (0.50, 0.82)	0.52 (0.50, 0.62)	0.55 (0.50, 0.85)
IFN-γ (pg/mL)	0.29 (0.19, 0.42)	0.31 (0.16, 0.37)	0.28 (0.19, 0.46)	0.28 (0.22, 0.36)	0.37 (0.34, 0.44)	0.26 (0.16, 0.30)	0.43 (0.28, 0.55)	0.29 (0.18, 0.54)	0.44 (0.26, 0.53)
TNF-α (pg/mL)	2.22 ± 0.84	1.74 ± 0.14	2.38 ± 0.23	2.57 ± 0.81	3.04 ± 0.51	2.42 ± 0.17	3.73 ± 0.98	4.06 ± 0.47	3.62 ± 0.25
Cytokine profiles									
Th1 profile	−0.53 ± 1.22	−0.95 ± 0.46	−0.40 ± 0.33	0.10 ± 1.51	1.14 ± 1.20	−0.16 ± 0.38	−0.09 ± 1.30	0.55 ± 0.69	−0.33 ± 0.33
M1 profile	−1.88 (−2.68, −0.72)	−2.51 (−3.57, −1.70)	−1.76 (−2.67, −0.47)	−0.31 (−1.48, 0.58)	−1.47 (−1.47, 1.38)	−0.25 (−1.53, 0.52)	1.85 (1.13, 3.27)	2.20 (1.74, 3.23)	1.83 (0.39, 3.25)
proinflammatory profile	−2.44 ± 3.31	−4.15 ± 0.99	−1.83 ± 0.93	−0.14 ± 2.53	1.93 ± 1.70	−0.52 ± 0.74	1.01 ± 2.60	1.70 ± 1.39	0.84 ± 0.79
anti-inflammatory profile	−0.35 (−1.55, 0.18)	−0.30 (−0.36, −0.04)	−0.74 (−1.81, 0.20)	0.84 (−0.32, 2.93)	1.93 (0.28, 3.63)	0.84 (−0.38, 2.52)	−0.91 (−1.78, −0.34)	−0.97 (−1.05, −0.72)	−0.85 (−1.92, −0.19)
Cytokine ratios									
Th1/Th2 ratio	−0.23 ± 1.24	−0.93 ± 0.33	0.01 ± 0.34	−0.43 ± 0.95	0.03 ± 0.76	−0.51 ± 0.28	0.46 ± 1.27	1.11 ± 0.82	0.21 ± 0.27
Th1/Treg ratio	−0.03 ± 1.00	−0.55 ± 0.56	0.15 ± 0.23	−0.36 ± 1.08	0.32 ± 0.58	−0.52 ± 0.31	0.33 ± 1.44	1.09 ± 0.72	0.01 ± 0.38
Th1/Th2+ Treg ratio	0.28 ± 1.21	−0.54 ± 0.52	0.55 ± 0.29	−0.75 ± 0.98	0.01 ± 0.63	−0.87 ± 0.28	0.95 ± 1.33	1.65 ± 0.81	0.66 ± 0.30
Proinflammatory/ anti-inflammatory ratio	−1.78 ± 2.82	−3.74 ± 1.02	−1.07 ± 0.73	−0.59 ± 1.82	1.85 ± 1.30	−1.03 ± 0.45	1.66 ± 2.52	2.49 ± 1.38	1.43 ± 0.79

ASD—autism spectrum disorder; ADHD—attention deficit/hyperactivity disorder; IL — interleukin; IFN-γ—interferon-gamma; TNF-α—tumor necrosis factor-alpha; Th—T helper lymphocytes; M1—macrophages M1; Treg—T regulatory lymphocytes. Data are expressed as mean ± SEM or median (interquartile range). ^a^ indicates the control group; ^b^ indicates the control females; ^c^ indicates the control males; ^d^ indicates the whole ASD group; ^e^ indicates the ASD females; ^f^ indicates the ASD males; ^g^ indicates the whole ADHD group; ^h^ indicates the ADHD females; ^i^ indicates the ADHD males.

**Table 4:** Comparison of circulating cytokines, cytokine profiles, and cytokine ratios between individual groups (i.e., ASD, ADHD, and control groups) with respect to sex.

Parameter	*p*-Value/ Cohen’s d a vs. d	*p*-Value/ Cohen’s d a vs. g	*p*-Value/ Cohen’s d d vs. g	*p*-Value/ Cohen’s d b vs. e	*p*-Value/ Cohen’s d b vs. h	*p*-Value/ Cohen’s d e vs. h	*p*-Value/ Cohen’s d c vs. f	*p*-Value/ Cohen’s d c vs. i	*p*-Value/ Cohen’s d f vs. i	*p*-Value/ Cohen’s d b vs. c	*p*-Value/ Cohen’s d e vs. f	*p*-Value/ Cohen’s d h vs. i
Cytokines												
IL-1α (pg/mL)	**0.025**/0.862	0.121/−0.089	**0.010**/−0.951	0.221/4.734	0.999/0.636	0.372/−4.098	0.477/0.446	0.145/−0.393	0.051/−0.839	0.999/−0.098	0.109/4.190	0.987/0.931
IL-1β (pg/mL)	**0.005**/0.776	**<0.001**/1.278	0.571/0.502	0.572/1.076	0.095/1.014	0.976/−0.062	0.080/0.683	**0.034**/1.338	0.890/0.654	0.803/−0.169	0.999/0.224	0.999/−0.492
IL-2 (pg/mL)	**0.043**/1.118	0.086/−0.342	**<0.001**/−1.459	0.522/2.770	0.936/0.256	0.522/−2.515	0.695/0.750	0.056/−0.588	**0.002**/−1.339	0.755/−0.348	0.901/1.671	0.275/0.495
IL-4 (pg/mL)	**0.021**/0.921	0.752/−0.382	**<0.001**/−1.303	0.883/1.297	0.999/−0.484	0.159/−1.781	0.579/0.805	0.999/−0.334	0.052/−1.390	0.999/0.075	0.999/0.567	0.999/−0.075
IL-6 (pg/mL)	**<0.001**/1.242	**<0.001**/1.638	0.152/0.396	0.241/2.351	0.221/2.092	0.999/−0.259	**0.021**/0.942	**<0.001**/1.668	0.257/0.726	0.987/0.120	0.676/1.528	0.999/0.544
IL-8 (pg/mL)	**0.010**/0.951	0.562/−0.205	**0.002**/−1.157	0.140/2.157	0.995/0.226	0.684/−1.932	0.516/0.618	0.921/−0.363	**0.048**/−0.980	0.993/−0.353	0.180/1.187	0.999/0.236
IL-10 (pg/mL)	**<0.001**/1.421	0.999/0.242	**0.007**/−1.179	0.352/2.169	0.989/−0.088	0.241/−2.257	**0.028**/1.201	0.999/0.371	0.314/−0.830	0.995/0.089	0.755/1.058	0.994/−0.369
IFN-γ (pg/mL)	0.935/−0.094	0.230/0.645	0.149/0.739	0.925/0.578	0.999/0.299	0.997/−0.279	0.939/−0.318	0.642/0.762	0.106/1.080	0.995/−0.193	0.452/0.703	0.990/−0.656
TNF-α (pg/mL)	0.629/0.401	**<0.001**/1.717	**<0.001**/1.316	0.302/1.515	**0.001**/2.687	0.999/1.172	0.108/0.039	**0.004**/1.435	**0.005**/1.395	0.999/−0.750	0.999/0.726	0.999/0.503
Cytokine profiles												
Th1 profile	0.513/0.475	0.934/0.332	0.999/−0.142	0.523/1.587	0.999/1.138	0.999/−0.448	0.999/0.180	0.999/0.046	0.999/−0.134	0.999/−0.419	0.999/0.987	0.999/0.673
M1 profile	**0.011**/1.210	**<0.001**/2.300	**0.011**/1.090	0.221/1.703	0.221/2.865	0.885/1.162	0.226/1.044	**<0.001**/2.097	0.082/1.054	0.917/−0.381	0.999/0.278	0.975/0.386
proinflammatory profile	0.098/0.795	**0.004**/1.193	0.896/0.399	0.226/2.123	0.116/2.045	0.999/−0.078	0.999/0.458	0.341/0.931	0.999/0.474	0.999/−0.811	0.999/0.854	0.999/0.302
anti-inflammatory profile	**0.002**/1.471	0.763/−0.056	**<0.001**/−1.527	0.684/1.645	0.621/−0.473	0.140/−2.118	**0.032**/1.422	0.999/0.091	0.053/−1.331	0.987/0.368	0.947/0.591	0.999/−0.197
Cytokine ratios												
Th1/Th2 ratio	0.999/−0.173	0.249/0.575	0.123/0.748	0.999/0.828	0.121/1.752	0.999/0.924	0.999/−0.445	0.999/0.169	0.999/0.614	0.999/−0.807	0.999/0.467	0.999/0.777
Th1/Treg ratio	0.999/−0.276	0.999/0.299	0.334/0.575	0.999/0.759	0.436/1.425	0.999/0.666	0.999/−0.582	0.999/−0.121	0.999/0.462	0.999/−0.607	0.999/0.734	0.999/0.939
Th1/Th2+Treg ratio	0.053/−0.857	0.288/0.560	**<0.001**/1.417	0.999/0.478	0.065/1.900	0.999/1.421	**0.038**/−1.239	0.999/0.093	**0.032**/1.332	0.999/−0.945	0.999/0.772	0.999/0.862
Proinflammatory/anti-inflammatory ratio	0.569/0.480	**<0.001**/1.383	0.071/0.903	0.107/2.372	**0.012**/2.646	0.999/0.273	0.999/0.020	0.178/1.022	0.285/1.043	0.534/−1.133	0.999/1.220	0.999/0.451

ASD—autism spectrum disorder; ADHD—attention deficit/hyperactivity disorder; IL — interleukin; IFN-γ—interferon-gamma; TNF-α—tumor necrosis factor-alpha; Th—T helper lymphocytes; M1—macrophages M1; Treg—T regulatory lymphocytes. A value of *p* ≤ 0.05 (in bold) was considered statistically significant. a indicates the control group; b indicates the control females; c indicates the control males; d indicates the whole ASD group; e indicates the ASD females; f indicates the ASD males; g indicates the whole ADHD group; h indicates the ADHD females; i indicates the ADHD males.

**Table 5:** Performance of autism spectrum disorder prediction using significantly changed complete blood count parameters and cytokines.

Parameter	Cut-Off Values	Sensitivity (%)	Specificity (%)	AUC
WBC	7.64	47.37	95.00	0.680
Mon	0.66	42.11	100.00	0.724
IL-1α	0.24	61.11	94.44	0.753
IL-1β	1.03	72.22	80.00	0.797
IL-2	2.95	42.11	95.00	0.725
IL-4	2.06	63.16	72.22	0.687
IL-6	0.69	73.68	90.00	0.857
IL-8	3.89	77.78	75.00	0.776
IL-10	0.70	80.00	75.00	0.839
M1 profile	−0.617	76.47	70.00	0.800
anti-inflammatory cytokine profile	0.023	68.42	90.00	0.808
combination of all significantly changed	−1.476	88.24	85.00	0.909

AUC—area under the curve; WBC—white blood cells; Mon—monocytes; IL—interleukin; M1—macrophages M1.

**Table 6:** Performance of autism spectrum disorder prediction in males using significantly changed parameters.

Parameter	Cut-Off Values	Sensitivity (%)	Specificity (%)	AUC
Mon	0.58	64.29	92.86	0.832
IL-6	0.69	71.43	93.33	0.843
IL-10	0.70	73.33	80.00	0.827
anti-inflammatory cytokine profile	0.67	60.00	100.00	0.822
Th1/Th2+Treg ratio	−2.96	100.00	0.00	0.144
combination of all significantly changed	−0.32	68.06	64.00	0.682
combination of significantly changed without Th1/Th2+Treg ratio	−0.32	75.00	76.67	0.802

AUC—area under the curve; Mon—monocytes; IL—interleukin; Th—T helper lymphocytes; Treg—T regulatory lymphocytes.

**Table 7:** Performance of attention deficit/hyperactivity disorder prediction using significantly changed complete blood count parameters and cytokines.

Parameter	Cut-Off Values	Sensitivity (%)	Specificity (%)	AUC
PDW	17.2	65	100	0.774
IL-1β	1.25	60	100	0.851
IL-6	0.86	94.12	95	0.988
TNF-α	2.89	85	80	0.893
M1 profile	0.187	94.12	95	0.985
proinflammatory profile	−0.007	73.33	68.42	0.761
proinflammatory/anti-inflammatory ratio	−0.667	85.71	68.42	0.782
combination of all significantly changed	0.193	92.68	73.68	0.887

AUC—area under the curve; PDW—platelet distribution width; IL—interleukin; TNF-α—tumor necrosis factor-alpha; M1—macrophages M1.

**Table 8:** Performance of attention deficit/hyperactivity disorder prediction in females using significantly changed parameters.

Parameter	Cut-Off Values	Sensitivity (%)	Specificity (%)	AUC
TNF-α	2.97	100	100	1
proinflammatory/anti-inflammatory ratio	0.02	100	100	1
combination of all significantly changed	0.02	100	100	1

AUC—area under the curve; TNF-α—tumor necrosis factor-alpha.

**Table 9:** Performance of attention deficit/hyperactivity disorder prediction in males using significantly changed parameters.

Parameter	Cut-Off Values	Sensitivity (%)	Specificity (%)	AUC
IL-1β	1.25	66.67	100	0.820
IL-6	0.67	100	93.33	0.990
TNF-α	3.23	73.33	86.67	0.836
M1 profile	0.03	92.86	93.33	0.971
combination of all significantly changed parameters	0.03	83.05	83.33	0.913

AUC—area under the curve; IL—interleukin; TNF-α—tumor necrosis factor-alpha; M1—macrophages M1.

**Table 10:** Overview of all evaluated peripheral immune markers in adolescents with autism spectrum disorder and attention deficit/hyperactivity disorder in comparison to control subjects in respect to sex.

Evaluated Parameters	ASD Adolescents	ASD Females	ASD Males	ADHD Adolescents	ADHD Females	ADHD Males
WBC	↑	=	=	=	=	=
Neu	=	=	=	=	=	=
Lym	=	=	=	=	=	=
Mon	↑	=	↑	=	=	=
PLT	=	=	=	=	=	=
MPV	=	=	=	=	=	=
PDW	=	=	=	↑	=	=
NLR	=	=	=	=	=	=
PLR	=	=	=	=	=	=
LMR	=	=	=	=	=	=
MLR	=	=	=	=	=	=
PMR	=	=	=	=	=	=
MPVLR	=	=	=	=	=	=
MPVPR	=	=	=	=	=	=
IL-1α	↑	=	=	=	=	=
IL-1β	↑	=	=	↑	=	↑
IL-2	↑	=	=	=	=	=
IL-4	↑	=	=	=	=	=
IL-6	↑	=	↑	↑	=	↑
IL-8	↑	=	=	=	=	=
IL-10	↑	=	↑	=	=	=
TNF-α	=	=	=	↑	↑	↑
IFN-γ	=	=	=	=	=	=
Th1 profile	=	=	=	=	=	=
M1 profile	↑	=	=	↑	=	↑
Proinflammatory cytokine profile	=	=	=	↑	=	=
Anti-inflammatory cytokine profile	↑	=	↑	=	=	=
Th1/Th2 ratio	=	=	=	=	=	=
Th1/Treg ratio	=	=	=	=	=	=
Th1/Th2+Treg ratio	=	=	↓	=	=	=
Proinflammatory/anti-inflammatory ratio	=	=	=	↑	↑	=

ASD—autism spectrum disorder; ADHD—attention deficit/hyperactivity disorder; WBC—white blood cells; Neu—neutrophils; Lym—lymphocytes; Mon—monocytes; PLT—platelets; MPV—mean platelet volume; PDW—platelet distribution width; NLR—neutrophils to lymphocytes ratio; PLR—platelets to lymphocytes ratio; LMR—lymphocytes to monocytes ratio; MLR—monocytes to lymphocytes ratio; PMR—platelets to monocytes ratio; MPVLR—MPV to lymphocytes ratio; MPVPR—MPV to platelets ratio; IL—interleukin; TNF-α—tumor necrosis factor-alpha; IFN-γ—interferon-gamma; Th—T helper lymphocytes; M1—macrophages M1; Treg—T regulatory lymphocytes. ↑ indicates increased levels of the evaluated parameter; ↓ indicates decreased levels of the evaluated parameter; = indicates unchanged levels of the evaluated parameter when compared to controls.

## Data Availability

Data are available upon reasonable request from the corresponding author.
